# Effects of slow dynamic, fast dynamic, and static stretching on recovery of performance, range of motion, balance, and joint position sense in healthy adults

**DOI:** 10.1186/s13102-024-00841-5

**Published:** 2024-08-09

**Authors:** Abdolhamid Daneshjoo, Elham Hosseini, Safoura Heshmati, Mansour Sahebozamani, David George Behm

**Affiliations:** 1 Department of Sports Injuries and Corrective Exercises, Faculty of Sports Sciences, Shahid Bahonar University of Kerman, Kerman, 7616913439 (ad) Iran; 2 School of Human Kinetics and Recreation, Memorial University of Newfoundland, St. John’s, NL A1C 5S7 Canada

**Keywords:** Recovery strategies, Knee fatigue, Sport injury

## Abstract

**Introduction:**

Considering the effects of fatigue on athletic performance and the subsequent increase in the probability of injury, the purpose of this study was to compare the effects of slow dynamic, fast dynamic, and static stretching on the recovery of performance, range of motion (ROM), balance, and joint position sense.

**Methods:**

Fifteen collegiate healthy females were involved in four separate sessions of slow dynamic stretching (SDS), fast dynamic stretching (FDS), static stretching (SS), and control condition (CC; without stretching), in a random order with at least 48 h of rest between sessions. After warming up, the individuals performed ROM, balance, joint position sense (JPS) maximum voluntary isometric contraction (MVIC) force as well as countermovement (CMJ) and squat jump (SJ) as pre-tests. After performing the knee fatigue protocol of 4 sets of knee extension and flexion at 60% of 1 repetition maximum (RM) to exhaustion (CC; without stretching) or stretching programs (SDS or FDS or SS), the subjects repeated all the tests at post-test 1 (after 5 min) and post-test 2 (after 60 min).

**Results:**

A significantly lower JPS error was detected with SDS while JPS error increased in the SS and control conditions (*p* < 0.0001). MVIC force significantly increased with SDS and FDS but decreased in control and SS conditions (*p* < 0.0001). Moreover, a significant decrease in CMJ and SJ height in SS and control conditions was revealed (*p* < 0.0001). Also, a significant decrease in balance with the control condition was revealed. But only SDS minimized fatigue-induced balance decrements (*p* < 0.0001). Additionally, the control condition experienced a significant decrease in knee extensor ROM, which contrasted with the significant increase in the quadriceps flexibility with the stretching conditions.

**Conclusions:**

The present results support the idea that SDS may increase quadriceps MVIC force, knee extensor ROM and knee JPS. So according to the present results, it is suggested that the SDS could be implemented and incorporated into a regular recovery program.

## Introduction

Although the health advantages of regular physical activity and sports are well known, exercise can cause muscular injury and fatigue [1]. Fatigue across training and competition periods increases injury risk, especially with high intensity training loads [2, 3]. Considering the relevance of fatigue and its effects on non-contact risk injuries (e.g., hamstrings strain injury, anterior cruciate ligament tear), several recovery strategies have been proposed to reduce the consequences of fatigue and improve sport performance, such as foam rolling, massage, and stretching [2, 4].

Stretching techniques are used to increase joint range of motion (ROM) [5, 6]. and they can assist in the prevention of musculotendinous injuries in activities of daily life or athletics [7]. Flexibility is a joint's capacity to move through its whole ROM. It has been specifically hypothesized that flexibility training's enhanced muscle–tendon compliance may increase elasticity and result in a stronger contraction force [8]. There are different types of stretching exercises, including static (SS) and dynamic stretching (DS) exercises, which are most commonly utilized among athletes and coaches [9, 10]. SS includes the limb moving to the end of its ROM and holding the stretched posture. SS traditionally has been the most popular stretching technique among athletes, because of its simple technique [11]. Nevertheless, some studies have found that prolonged SS can reduce strength, power or endurance up to 20.5% [12]. whereas DS may have no effect or improve subsequent muscle strength performance [13, 14]. As a result, DS has been recommended as a replacement to SS because it can improve agility, endurance, strength, power, and anaerobic capacity [15].

In DS, motions are performed at or near full ROM under controlled situations at slow to relatively fast velocity [5]. DS improves proprioception, muscle strength, and performance improvements by increasing the neuronal activity of the motor unit and improving kinesthetic awareness [16]. Additionally, researchers proposed that DS can maintain or enhance the stiffness of muscle-tendinous units (MTUs), boost nerve impulse transmission, and increase force output at higher velocities (force–velocity relationship) [17]. The extent of the stretch-induced effects depends on various variables, including muscle condition, stretching time, stretching intensity, contraction type, and contraction velocity. However, these mechanisms have not yet been evaluated, and it is still unclear how DS affects performance [16–18].

Accordingly, past research findings regarding the influence of SS and DS on muscle function and performance are conflicting. For example, recent studies have demonstrated that acute prolonged bouts of SS (> 60-s per muscle group) can impair vertical jumps, short sprints, tasks requiring maximal voluntary contractions, muscle strength-endurance performance, balancing tasks, and reaction time [12, 19, 20]. Nevertheless, these detrimental impacts of SS training are dependent on the time and intensity of stretching. Accordingly, Behm and Chaouachi [21]. recommended avoiding SS of any duration when even small performance reductions are unacceptable as with elite competitions. This suggestion might only apply to SS [21]. as studies have found that DS has trivial negative impacts on strength and power output [22]. sprint performance [23]. and vertical jump [24]. Recent research has demonstrated that DS increases muscle strength, jump height, and sprint efficiency [25]. However, the literature suggests that shorter durations of DS do not negatively affect performance and that longer durations (20–30 s) may improve performance [15, 18]. Also, DS may impair strength at slow velocity but enhances it at faster velocities [26]. Therefore, there is a lack of consensus in the literature about the effects of dynamic stretching (DS) and static stretching (SS) on muscular performance, functional abilities, and reducing risk of injury..

According to past literature, with the onset of fatigue, the athlete’s strength, performance, and neuromuscular coordination are reduced, and the athlete is more exposed to injury. Therefore, researchers have proposed different methods, such as different types of stretching for recovery, improving the effects of fatigue and reducing the risk of injury. Regarding the literature of the effects of different stretching techniques on performance and durability in future competitions, contradictory and limited results have been reported. Then, we aimed to compare the effects of slow DS, fast DS, and SS on recovery of performance, ROM, balance, and joint position sense of healthy adults and to investigate the sustainability of these effects over a subsequent hour. The main hypothesis of the present study is that after fatigue, the intergroup effects In the stretching of slow DS, fast DS and SS, there is no significant difference on the recovery factors of function, balance and proprioception of healthy adults. The researchers are looking for a response to the question, "Which type of stretching is more useful in recovering sports performance?".

## Methods

### Ethics Statement

The participants were instructed about any possible risks related to the present procedures and they signed a written informed consent. The present study was approved by the local Ethical Committee of Shahid Bahonar University of Kerman (IR.UK.REC.1401.028). The authors followed the principles outlined in the Declaration of Helsinki.

### Participant

Fifteen collegiate healthy females (age 23.86 ± 2.57 years; height 164 ± 4.47 cm; mass 59.28 ± 7.09 kg), volunteered for the present study. Women with a body mass index (BMI) between 21 and 25 kg/m2, a history of lower limb injury, trauma, or disease within the previous six years, limited range of motion, and no participation in a lower limb stretching program were the inclusion criteria. The Physical Activity Readiness Questionnaire, or PAR-Q, (Thomas et al., 1992) was also used to assess health. The sample size of 15 physical education students was determined using the G*Power software (Version 3.1.9.4) (repeated measure ANOVA, α = 0.05, ES = 0.40) with the statistical power of 0.9 [27]. The participants were involved in four separate sessions of slow dynamic stretching (SDS), fast dynamic stretching (FDS), static stretching (SS), and control condition (CC; without stretching). The four sessions were separated by 48 h and their order was randomized [28, 29]. The participants avoided any form of intense physical activities at least two days before testing sessions. Also, during this period, participants abstained from alcohol and caffeine. Generally, participants were recreationally active in several sports but were not engaged in any official competition or regular intensive physical activities at that time of the study. Participants with lower extremity joints or muscular diseases were excluded from study. The regular use of drugs as well as the presence of any cardiovascular and pulmonary diseases or who had participated in massage, foam roller, hydrotherapy protocol, or had heavy physical activities in 24-h prior the test (based on their self–reports and medical records) were considered as exclusion criteria.

### Procedure

**Fig. 1:**
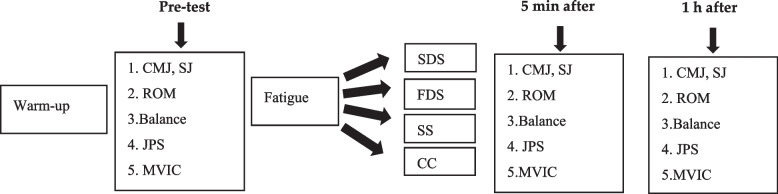
Overview of the test procedure (CMJ = counter movement jump, SJ = Sargent jump, ROM, range of motion, JPS = joint position sense, CC = control condition, h = hour, min = minute)

Forty-eight (48) hours prior to the testing procedure, all participants attended a 2-h familiarization session to introduce the tests and complete the personal and consent forms. Then, height, and body mass were measured. Additionally, the dominant leg, which was determined by asking the participants which leg they would predominantly use to kick a ball, was designated as the test leg [30]. One-repetition maximum (1RM) was measured by equation; 1RM = Weight/1 − 0.02 (Rep) (3). At each session after a 5-min warm-up exercise of between 70–80 revolutions per minute (RPM) at an intensity of 1 kilopond (Monark Exercise, 839 E, Sweden) [31]. participants performed tests in a random order. Measures consisted of strength and power performance (vertical jumps and maximal voluntary isometric contraction (MVIC) force / strength), ROM (flexibility), balance and joint position sense (JPS). Subsequently, participants performed a fatigue protocol (4 sets of knee extensions and knee flexion at 60% of 1 repetition maximum (RM) to exhaustion), which was followed in a randomized order by SDS, FDS, SS, and CC conditions and then performed post-tests after 5-min and 1 h (Fig. 1) [32]. During the testing process, conditions for all participants, including lighting, temperature and noise, were the same for all participants. We encouraged all participants to maintain similar eating habits and sleeping patterns. Testing measures took approximately 30-min to complete. The assessments were performed over four experimental sessions, with each session being spaced apart by a duration of 48 to 72 h [28, 29]. Each testing measurement was supervised by one of the researchers (who was blinded to each participants stretching condition) and conducted between 8 and 11 am.

**Fig. 2:**
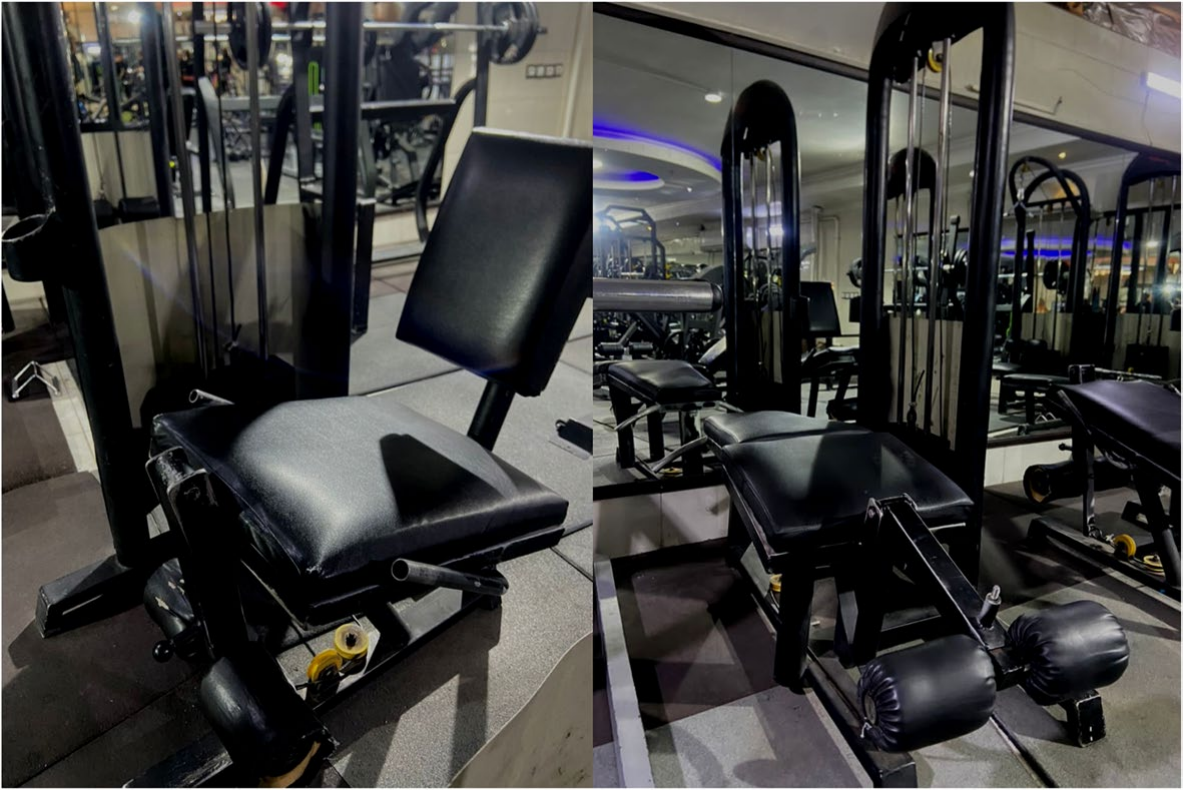
Fatigue protocols with knee extension machine and lying leg curl machine

The fatigue protocol in this study included 4 sets with 60% 1RM knee extension with a knee extension machine for quadriceps and 4 sets with 60% 1RM knee flexion movement with a Lying Leg Curl machine for the hamstrings (Fig. 2). In addition, between each set, a three-minute rest period was provided [33]. Fatigue in this study was operationally defined as the point at which participants were unable to perform full knee flexion and extension. Additionally, the Rating of Perceived Exertion (RPE) was assessed as an indicator reflecting the intensity of training, with a target RPE score exceeding 17 signifying that the participants perceived the training as "very hard." More specifically, at the end of each set, the person was considered exhausted when they could no longer complete a full knee flexion and extension repetition of movement (which meant their ROM had decreased by more than 30%) and gave the exercise a score of 17 on the RPE scale [34, 35].

### Stretching protocols

After the fatigue protocol, the stretching for the hamstrings included standing hamstrings stretch (standing position with the feet hip-width apart and extended knees, the participants flexed forward at the hips, lower their head toward the floor and finally wrapping their arms around lower legs) and lift the leg up (from the initial standing position, one leg with the knee fully extended is flexed from the hip joint and moves towards the trunk). The stretching for the quadriceps included standing quadriceps stretches (The subject stands on one leg and flexes the contralateral knee and maintains balance with one hand on the wall, then moves the flexed knee back as far as possible. If necessary, to increase the knee flexion stretch, the hand slowly pulls the heel towards the gluteal muscles.) and forward lunges (a large forward stride with the feet hip-width apart; the pelvis is lowered toward the floor; and the anterior knee is bent as much as possible). The same exercises were used in all stretching protocols (SDS, FDS and SS), but in the SDS and FDS protocols, muscles were stretched using the dynamic mode, while in the SS regime the exercises were performed in static positions after reaching the maximum amplitude [36]. With SDS and FDS, each movement was performed for 30 s, in which six repetitions were completed. FDS was performed at 100 beats/min and SDS at 50 beats/min [17]. The rest between sets and exercises (6 sets of 30 s) was 10-s as well. The total duration of the entire stretching exercises protocol was approximately 7:30-min. Furthermore, all participants rested for 5-min between the stretching protocol and measurements, also in the non-stretching (control) session participants rested for 5-min between the fatigue protocol and measurements.

### Vertical Jumps

The countermovement jump (CMJ) started with the participants standing in an upright position, hands on the hips to avoid the contribution of the arms to the jumping performance. The CMJ involved a rapid downward movement of the knee to approximately 90° flexion, followed by a quick vertical upward movement as high as possible, all in one sequence. Squat jump (SJ) was used to measure leg strength in the concentric mode, with the movement starting from a static 90° knee flexion position. This study used the Jump-and-Reach Method with wall tape to assess jump height. This approach has been introduced for field evaluation in addition to its relative look among researchers. Because this approach provides the desired outcome more quickly and with less equipment [37]. (CMJ; ICC = 0.98, SJ; ICC = 0.97) [38].

### Knee Extensors and Flexors MVIC Force

A hand-held dynamometer (HHD) (Lafayette manual muscle testing system model 01163; Lafayette Instrument Company, Lafayette, IN) was used to test the dominant leg. The intra-rater reliability of HHD is good to excellent (ICC = 0.80–0.96) [39]. A standard therapy bed was used with an unstretched strap to attach the dynamometer to the leg.. HHD was fixed with a rigid belt perpendicular to the ankle (five cm above the malleoli), with a pad between the tibia and the dynamometer to reduce the discomfort caused by the contact. Participants completed three MVIC for 5-s, with a 60-s rest after each trial. Participants held their arms onto the chest to stabilize their trunk and pelvis. The participants performed two submaximal efforts as a familiarization trial and then performed knee extension and knee flexion two times with 1-min rest interval [40]. The tests were conducted for quadriceps muscle while participants sitting on a leg extension machine and hamstrings muscle strength were evaluated in a prone position. In both tests, the strap was secured to limit knee flexion to 85 degrees during extensor and flexor contractions [41]. The maximum force in kilograms was recorded for each test [42].

### Dynamic balance test

Dynamic balance was evaluated using the Y-balance test that is performed on a grid of three lines. Test–retest reliability were reported moderate to excellent for the right limb (ICC 0.681- 0.908) and moderate to good for left limb (ICC 0.714—0.811) [43]. The foot of the dominant leg was positioned in the center of the grid, so that the foot was bisected equally in the anteroposterior and medial–lateral planes. It was performed by pushing the prepared wooden box with the tip of the foot in the anterior, posterior, and postero-lateral directions (as far as possible along the designated line), using an instrument composed of the Y-balance test form on the floor [44]. Participants were asked to keep their hands on their iliac crests and to keep the heel of their stance leg on the ground and avoid using the reaching leg for a substantial amount of support at any time through the trials. The sum of all measured values was divided by two times the participant's limb length (limb length was measured from the anterior superior iliac spine (ASIS) to the distal end of the medial malleolus (cm)), multiplied by 100, and divided into the composite scores for the analysis (excursion distance/leg length) × 100 = %maximum reach distance) [44, 45]. The higher excursion distance reflected the greater dynamic balance.

### Range of Motion (ROM)

The sit-and-reach test (SR) was used to evaluate trunk and lower extremity ROM. The SR has shown to have a high intra-class correlation coefficient, (ICC = 0.98) [46].The Baseline® (Cooper Institute/YMCA, AAHPERD) device was used for evaluation. The participants were instructed to place their heels on the device while in the sitting position with their trunk flexed at 90° flexion. After the participants’ arm length were determined by the device, they were asked to push the device with their fingertips without raising their knees and reach forward as far as possible. Participants slowly pushed the indicator forward as far as possible. The measurement was performed three times. The average of the results was recorded as the result of the SR test [47].

### Knee flexion range of motion (ROM)

Knee flexion ROM was measured as the participant performed the modified Thomas test (ICC = 0.98) [48]. First, reflective markers were adhered to participants’ skin or tight-fitting garments on the lateral femoral epicondyle, greater trochanter, and lateral malleolus. Once placed, the markers were not removed until after the final testing procedure. All digital photographs were taken with a Canon camcorder (MV750i 8 megapixels). The tripod height was set at 92 cm, and the camera was fully zoomed-out and positioned exactly 1.8 m perpendicular to the end of a non-adjustable 76 cm high examination Table [49]. It was performed by having the participant hold his or her non-testing knee (non-dominant leg) to his or her chest, while letting the thigh and leg of the testing hip (dominant leg) hang freely. Each participant completed the test three times. Between each trial, the participant was asked to stand up from the table. The average of each participant's three trials was then used for analyses [50].

### Joint position sense (JPS)

Knee JPS was measured using digital photography with a Canon camcorder (MV750i 8 megapixels). The reliability of this measurement method with AutoCAD software has been reported as high (ICC = 0.97) [51]. Participants were prepared for assessment with application of adhesive markers on the greater trochanter of the dominant leg, lateral tibiofemoral joint line and lateral malleolus [52]. A goniometer was fixed at 45° knee flexion on a bar next to the participant. Participant sat on the chair, then examiner passively extends the knee joint from the starting position (starting knee angle of 90° flexion) to the target angle of 45° at a very slow speed [53]. After holding the leg in this position for 5-s, the examiner returned the leg to the starting position. The participants were then asked to actively reproduce the same knee angle that was passively positioned by the examiner [30, 54]. During the experiments, participants were blindfolded and wore headphones to eliminate visual and auditory cues. The mean joint positioning error of the two measurement degrees of error from the target position were recorded for analysis. Lower mean error scores indicated the better knee JPS [51].

### Statistical Analyses

Statistical analysis was performed using the SPSS Version 26 (Armonk, NY: IBM Corp.). The Levene's and Shapiro–Wilk tests were employed for assessing homogeneity of variance among conditions and normality of the distribution of scores (*p* > 0.05). A 2-way mixed repeated measure ANOVA with time (pre–test vs. 5-min vs. 1-h) and conditions (SDS vs. FDS vs. SS vs. CC); as factors was performed for all dependent variables. When condition-time interactions were observed, the post-hoc Bonferroni test was conducted to identify pairwise differences The effect size was assessed by partial eta squared, and considered as either small (pη2 = 0.01), medium (pη2 = 0.06), or large (pη2 = 0.14) [27]. A significant level was accepted at *p*-value < 0.05 for all statistical parameters.

## Results

### Countermovement Jump (CMJ) Height

The results showed significant condition and time interactions (F_6,112_ = 4.49, *p* < 0.0001, pη2 = 0.19) with significant jump height decreases in the CC 5-min (*p* = 0.001, 14.5%) and 1-h (*p* = 0.009, 2.1%) after the fatigue protocol. The results showed significant decreases in jump height after SS among times (F_2,112_ = 7.93, *p* = 0.001, pη2 = 0.12). Moreover, significant jump height decreases at 5-min (p = 0.017, 7.9%) after SS was revealed. But the results did not show any main effects for conditions (F_3,56_ = 0.880, *p* = 0.457), (Table 1).

**Table 1:** Vertical jumping, and maximal voluntary isometric contraction (MVIC) strength of knee muscles among control, SDS, FDS and SS stretching groups (values are mean ± SD)

	Group	Pre-test	5-min	1-h
Countermovement jump height	Control	28.9 ± 4.1	24.7 ± 3.9*	28.3 ± 4.1*
(cm)	SDS	28.9 ± 4.6	29.8 ± 4.6	29.8 ± 4.2
	FDS	28.7 ± 4.1	28.4 ± 3.8	28.3 ± 3.6
	SS	29.1 ± 4.7	26.8 ± 3.5*	28.1 ± 4.2
Squat jump height (cm)	Control	27.7 ± 2.9	24.9 ± 3.7*	27.9 ± 5.1
	SDS	28.3 ± 4.2	29.1 ± 3.9	29.3 ± 5.4
	FDS	27.9 ± 2.6	28.5 ± 4.4	28.5 ± 4.3
	SS	28.3 ± 3.8	26.1 ± 3.2*	27.9 ± 5.0
Hamstrings MVIC strength	Control	24.1 ± 3.1	20.7 ± 3.5*	23.1 ± 3.3
	SDS	24.4 ± 3.1	26.7 ± 2.8^a^	24.4 ± 5.1
	FDS	24.0 ± 3.1	23.6 ± 3.3	23.4 ± 6.2
	SS	23.6 ± 2.9	20.7 ± 3.5*	22.3 ± 3.4
Quadriceps MVIC strength	Control	35.8 ± 3.8	30.8 ± 4.1*	32.7 ± 4.4
	SDS	36.7 ± 3.0	38.3 ± 3.0^a^	36.1 ± 3.6
	FDS	36.5 ± 3.1	36.7 ± 4.2^a^	35.4 ± 6.2
	SS	36.4 ± 2.5	33.9 ± 3.5*	35.2 ± 4.4

*SDS *Slow dynamic stretching group, *FDS *Fast dynamic stretching group, *SS *Static stretching group, * = significant difference with pre-test (*p* < 0.05), ^a^ = significant difference with control group (*p* < 0.05)

### Squat Jump (SJ) Height

Significant interactions between condition and time (F_6,110_ = 3.74, *p* = 0.002, pη2 = 0.17) with decreases in jump height at 5-min after fatigue protocol in the CC (*p* = 0.002, 10.1%) and SS (*p* = 0.015, 7.8%). Main effect for time showed significant decreases in jump height (F_2,55_ = 4.68, *p* = 0.013, pη2 = 0.14). But the results did not show any main effect for conditions (F_3,56_ = 0.966, *p* = 0.415), (Table 1).

### MVIC of hamstrings and quadriceps

Significant interactions between condition and time for hamstrings (F_6,112_ = 8.14, *p* < 0.0001, pη2 = 0.31) and quadriceps MVIC strength (F_6,112_ = 6.86, *p* < 0.0001, pη2 = 0.27) were revealed. Significant decreases in CC 5-min after fatigue protocol in hamstrings (*p* = 0.001, 14.1%) and quadriceps (*p* = 0.001, 14.0%) MVIC strength were found. Moreover, significant decreases in hamstrings (*p* = 0.001, 12.3%) and quadriceps (*p* = 0.01, 6.9%) MVIC strength, 5-min after SS was found. The results show significant main effects for times for hamstrings (F_2,55_ = 6.03, *p* = 0.004, pη2 = 0.18) and quadriceps MVIC strength (F_2,55_ = 7.25, *p* = 0.002, pη2 = 0.21). The results show significant main effects for conditions in hamstrings (F_3,56_ = 3.40, *p* = 0.02, pη2 = 0.15) and quadriceps MVIC strength (F_3,56_ = 4.50, *p* = 0.007, pη2 = 0.19). Hamstrings MVIC strength following SDS was significantly greater than control (*p* = 0.035, ES = 1.9). Similarly, quadriceps MVIC strength was higher with SDS (*p* = 0.006, ES = 2.1) and FDS (*p* = 0.047, ES = 1.4) versus control (Table 1).

### Balance

The results showed significant interactions between condition and time (F_6,112_ = 6.32, *p* < 0.0001, pη2 = 0.25). The results showed significant decreases in balance among times (F_2,112_ = 12.52, *p* < 0.0001, pη2 = 0.18). Significant decreases in CC after 5-min (*p* = 0.001, 6.7%) and 1-h (*p* = 0.013, 3.9%) was found. But the results did not show any main effects between conditions (F_3,56_ = 0.286, *p* = 0.835) (Table 2).

**Table 2:** Flexibility, balance, and joint position sense among Control, SDS, FDS and SS stretching groups (values are mean ± SD)

	Group	Pre-test	5-min	1-h
Sit and Reach (cm)	Control	31.0 ± 6.6	29.0 ± 4.9	31.7 ± 5.9
	SDS	30.4 ± 6.4	33.7 ± 5.1*	32.5 ± 5.8*
	FDS	30.7 ± 6.2	32.7 ± 5.3	31.7 ± 5.6
	SS	31.6 ± 5.8	32.8 ± 4.2	32.9 ± 6.4
Modified Thomas test	Control	46.3 ± 5.6	41.9 ± 7.2*	44.6 ± 5.9
(cm)	SDS	47.4 ± 5.7	53.2 ± 6.4*^a^	52.8 ± 8.5
	FDS	46.9 ± 5.7	51.2 ± 7.2*	50.1 ± 7.6
	SS	47.6 ± 5.9	51.5 ± 8.4*	48.0 ± 9.7
Y Balance test (cm)	Control	99.3 ± 6.1	92.6 ± 6.3*	95.4 ± 6.3*
	SDS	98.2 ± 8.9	98.4 ± 9.0	97.4 ± 7.9
	FDS	99.2 ± 7.1	97.9 ± 7.6	96.6 ± 8.0
	SS	97.3 ± 6.1	97.5 ± 8.6	96.1 ± 7.8
Joint Position Sense (°)	Control	4.7 ± 1.4	7.3 ± 1.8*	5.6 ± 2.8
	SDS	4.9 ± 1.6	3.5 ± 1.9*^a^	3.9 ± 1.9
	FDS	4.4 ± 1.2	5.1 ± 2.1	4.5 ± 2.0
	SS	4.9 ± 1.5	5.5 ± 1.6*	5.8 ± 2.6

*cm *centimeter, ° degree, *ROM*  knee range of motion, *SDS *Slow dynamic stretching group, *FDS *Fast dynamic stretching group, *SS *Static stretching group, * = significant difference with pre-test (*p* < 0.05), ^a^ = significant difference with control group (*p* < 0.05)

### ROM (SR and Thomas tests)

The results showed significant interactions between condition and time in SR (F_6,112_ = 2.76, *p* = 0.016, pη2 = 0.13) and Thomas test (F_6,110_ = 6.82, *p* < 0.0001, pη2 = 0.27). The results showed significant main effect differences among times in SR (F_2,112_ = 3.84, *p* = 0.024, pη2 = 0.06) and Thomas (F_2,55_ = 7.25, *p* = 0.002, pη2 = 0.20) tests. Significant increase in SR scores in SDS after 5-min (*p* = 0.002, 10.8%), and 1-h (*p* = 0.017, 6.9%) were revealed. Significant increase of Thomas scores after 5-min in SDS (*p* = 0.001, 12.2%), FDS (*p* = 0.04, 9.2%), and SS (*p* = 0.037, 8.2%) were detected. A significant decrease in Thomas score in CC after 5-min (*p* = 0.004, 9.5%) was exposed. The results did not show any main effects between conditions in SR test (F_3,56_ = 0.36, *p* = 0.779). The results show significant main effect differences between conditions in Thomas test (F_3,56_ = 3.66, *p* = 0.018, pη2 = 0.16). SDS showed significantly (*p* = 0.014) greater ROM compared to control (Table 2).

### Joint Position Sense (JPS)

The results showed significant interactions between condition and time (F_6,110_ = 6.13, p < 0.0001, pη2 = 0.25). The results showed significant main effect differences in JPS among times (F_2,55_ = 3.43, *p* = 0.04, pη2 = 0.11). Significant increase of JPS errors in CC (*p* = 0.001, 55.3%) and SS (*p* = 0.03, 12.2%) after 5-min were found. Significant improvement in SDS (*p* = 0.045, 28.6%) after 5-min was found. The results show main effect differences between conditions (F_3,56_ = 4.77, *p* = 0.005, pη2 = 0.20). The differences between SDS with CC (*p* = 0.006) were significant (Table 2) with SDS showing less JPS errors.

## Discussion

The aim of this study was to investigate the effect of the SDS, FDS, and SS on recovery of jump height, MVIC strength, ROM, balance, and JPS of healthy collegiate girls. In accordance with the literature [9, 55]. this study showed that after a knee fatigue protocol, significant, large magnitude decreases, were observed in jump height, MVIC strength, dynamic balance, JPS and knee ROM in CC (control condition). Also, the results of present study showed that after 5-min SS led to a decrease in CMJ, SJ, MVIC, knee joint ROM (quadriceps) and JPS. It revealed that SDS was able to increase or recover the pre-test values of hamstring and quadriceps MVIC force, JPS, and knee extensors ROM after 5-min. Also, recovery of all factors to pre-test values were observed after 1-h (after the fatigue protocol) with SS, SDS, and FDS. Only SR scores after 1-h increased compared to pre-test after SDS condition.

*Five minutes following the fatigue protocol, all measures were significantly reduced in CC. The results showed that after 1-h of recovery, CMJ height and balance in CC still declined.* According to the literature fatigue decreases muscular power and disrupts the function of proprioceptive receptors, especially muscle spindles and Golgi tendon organs, reducing their sensitivity to neural impulses, therefore possibly increasing injury risk at the end of competitions [9, 55].

*Five minutes after SS the significant decrease in jump heights, MVIC knee strength, knee joint (quadriceps) ROM and JPS were still evident. But in SS condition after 1-h, the results did not show differences with pre-test.* The results suggest against using SS after a fatigue protocol to recover these factors after 5-min. The results by Robbins and Scheuermann [56]. into the effects of SS on vertical jump height demonstrated a decrease in jump height following the SS protocol [56]. The main causes may be a decrease in neuromuscular activation and musculotendinous stiffness [57]. Moreover, prior research demonstrated that acute bouts of SS could lengthen muscles, and alters the motor unit's viscoelastic characteristics [13]. Hence, a muscle may develop a less-than-optimal cross bridge overlap, reducing muscular strength. Also, the muscle length tension curve, speed of the sarcomere stretch–shortening cycle, and decreased muscle activation and reflex excitability could all be impacted by these alterations [13]. The results of the previous investigation were similar with the findings from the present study that, 30 s of SS decreased power parameters [21]. However contrary to the present findings, Holt and Lambourne [58]. observed no difference in vertical jump performance after 15 s of SS in male soccer players [58]. The possible reasons for the contradictory results with the present study are the different static stretching protocols (Holt and Lambourne performed SS of hamstrings, gluteals, lower back, quadriceps, and the hip flexors). Moreover, their subjects were male soccer players, and these differences with present study may be attributed to differences in participant sex and athletic history [59].

Earlier research revealed that an increased pain threshold due to stretching (sense of pain during joint movement), may enhance knee ROM among healthy males [60]. Hence, the relative change in muscle–tendon unit stiffness is related to SS intensity [61]. Moreover, Ghaffarinejad et al., [62]. reported that SS (30 s stretch followed by a 30 s pause) improves the sensitivity of muscle spindles and neuronal message transmission to the central nervous system among healthy males and females [62]. and this may contribute to the increased ROM after SS. Also Costa et al., [55]. reported that SS did not have a negative effect on balance beyond the fatigue protocol, but it did not reduce the negative consequences of fatigue [55]. A previous study showed that a 15 s moderate SS program can enhance dynamic balance performance by increasing postural stability. In addition, it was found that increased volumes of SS and DS result in partial balancing enhancements during the star excursion balance test [55]. The contrast with the findings of the present study may be related to the use of Biodex balance system to evaluate dynamic balance following SS.

According to Larsen et al., [63]. the static stretch protocol in healthy subjects had no impact on JPS [63]. The findings of Farshidi et al., [51]. were relatively consistent with the present study as they demonstrated that whereas SS can enhance JPS, PNF and DS had greater effects compared to SS [51]. SS can alter the sensitivity of muscle receptors [9]. (e.g., Golgi tendon organs and muscle spindles) affecting proprioception. On the other hand, the more inactive nature of SS is another factor that may have an impact on the findings of the current study. Contrary to dynamic stretching, muscular contractions are not required to enhance muscle flexibility with SS. The findings suggest that the SS has no impact on the muscles' proprioception receptors [9].

*The results revealed that SDS showed more improvement compared with no-stretching conditions after 5-min in hamstrings and quadriceps MVIC force, JPS, SR and knee extensors ROM. No differences in SS and FDS compared to control group except significant differences in quadriceps MVIC force after 5-min FDS*. These results support that SDS is a superior stretching technique to enhance the MVIC strength, JPS, SR and knee extensors ROM after 5-min than other stretching techniques. Previous research found that DS did not improve short-term explosive performance [64, 65]. although other studies reported improve jump performance [10, 21, 66]. Moreover, the literature has shown that after DS, EMG activity during a vertical jump task was increased [18]. In general, Behm and Chaouachi [21]. explained that DS techniques are an effective way to enhance explosive muscular contractions compared to SS. It has been hypothesized that increased body and muscle temperatures are among the mechanisms through which DS enhances muscular performance [21]. by increasing the rate of nerve impulses and sensitivity of nerve receptors. Moreover, after DS, an improvement in neuromuscular function has also been linked to improvements in reflex sensitivity [18]. In contrast, Opplert and Babault [18]. found that SS had no effect on presynaptic inhibition while DS resulted in a considerable decrease. The author stated that this reduction may be explained by the quick lengthening and contraction of the muscle fibers, which do not occur during SS [18].

Salekar et al., [67]. found that both DS and SS induced significant increases in hamstrings flexibility; however, DS was more efficient than SS. Overall, DS contracts the antagonist muscle and relaxes the lengthening muscle with reciprocal inhibition. Other researchers’ state that the slow build-up of tension and lack of soreness, reduce stretch reflex response, relaxing muscles and allowing a greater extent of stretching [18, 67]. Fletcher [17]. reported that the FDS demonstrated considerably higher jump height across all tests when compared to the SDS and no stretching conditions [17]. Although the causes of these performance changes are complicated, it seems that SDS are associated with increases in heart rate and core body temperature, whereas the FDS intervention is associated with greater nervous system activation. According to the authors' hypothesis, DS could maintain or enhance the stiffness of the musculotendinous unit (MTU) and improve nerve impulse transmission, resulting in positive changes to the force–velocity relationship. Additionally, they hypothesized that the major benefits of DS include greater sensory sensitivity and increased motor unit reflexes, which improve proprioception and pre-activation. The literature suggests that a faster dynamic stretch can improve athletic performance. However, it should be noted that all of these studies were conducted to find out the optimal warm-up, so the conclusions may not apply to recovery from fatigue [17]. The results of this study confirm the hypothesis that FDS increases fatigue and may negatively affect athlete’s performance. Therefore, the result of this study supports the hypothesis that SDS may enhance recovery after knee fatigue in MVIC strength, JPS and quadriceps’ flexibility in collegiate females. In other words, SDS may more effectively attenuate fatigue.

There are several limitations that need to be considered. First, the investigators were unable to perform the intervention for a longer period (e.g., two or three months). Second, the current study did not assess the stiffness of passive tendon and delayed onset muscle soreness (DOMS). Since SS is suggested as preferable when start a flexibility program with stiff hamstrings [8]. hamstrings tightness should be considered when deciding whether to include SS or DS in a training program.

The limited number of participants and the exclusive focus on women were identified as additional limitations of this research. Moreover, future research could address these limitations and investigate the long-term effects of SDS on sport performance and reducing the risk of injuries, also researchers could use the OMNI-RES scale for sensation of fatigue that described more precisely [68].

## Conclusion

It can be concluded that knee fatigue may decrease knee modifiable risk factors such as jump height, MVIC strength, dynamic balance, knee extensor ROM and JPS. The results of the present study support that SDS may induce increases in the MVIC strength, SR, quadriceps ROM and knee JPS after 5-min. According to the present results, it is suggested that the SDS could be implemented and incorporated into regular recovery program among collegiate females.

### Practical Applications

Considering the significant prevalence of knee injuries in female athletes, particularly after fatigue, this research is looking for the best method to reduce the negative effects of fatigue, maintain the athlete's performance, and potentially decrease the risk of injury. The results of the current study also revealed that after performing the fatigue protocol, the parameters of jump height, MVIC strength, dynamic balance, knee extensor ROM, and JPS significantly decreased. Therefore, a person who is exhausted will experience a decrease in performance in addition to an increase in their risk of injury. Therefore, it is crucial to determine an appropriate recovery strategy in order to maintain the health of the athlete to achieve the best possible result in competitive sports. The results of the research show that slow dynamic stretching (SDS) has the ability to significantly improve the parameters of Jump height, MVIC strength, SR, quadriceps ROM, and knee JPS after fatigue. Despite noticing that the parameters of dynamic balance and JPS reduced after performing the fast dynamic stretching (FDS) method, FDS was still able to have a positive impact on Jump height, Quadriceps MVIC, and ROM variables. But after performing static stretching (SS), other parameters decreased and the only impact was helpful on the ROM factor. This research generally recommends slow dynamic stretching at this time for use as a recovery technique.

## Data Availability

The data that support the findings of this study are available from the corresponding author, upon reasonable request. Because the data are not publicly available due to their containing information that could compromise the privacy of research participants.
